# Selective Auditory Attention Detection Using Combined Transformer and Convolutional Graph Neural Networks

**DOI:** 10.3390/bioengineering11121216

**Published:** 2024-11-30

**Authors:** Masoud Geravanchizadeh, Amir Shaygan Asl, Sebelan Danishvar

**Affiliations:** 1 Faculty of Electrical & Computer Engineering, University of Tabriz, Tabriz 51666-15813, Iran; amir.shaygan@tabrizu.ac.ir; 2 College of Engineering, Design and Physical Sciences, Brunel University London, London UB8 3PH, UK

**Keywords:** selective auditory attention detection, graph neural network, transformer, convolutional neural networks, brain connectivity, hybrid neural networks

## Abstract

Attention is one of many human cognitive functions that are essential in everyday life. Given our limited processing capacity, attention helps us focus only on what matters. Focusing attention on one speaker in an environment with many speakers is a critical ability of the human auditory system. This paper proposes a new end-to-end method based on the combined transformer and graph convolutional neural network (TraGCNN) that can effectively detect auditory attention from electroencephalograms (EEGs). This approach eliminates the need for manual feature extraction, which is often time-consuming and subjective. Here, the first EEG signals are converted to graphs. We then extract attention information from these graphs using spatial and temporal approaches. Finally, our models are trained with these data. Our model can detect auditory attention in both the spatial and temporal domains. Here, the EEG input is first processed by transformer layers to obtain a sequential representation of EEG based on attention onsets. Then, a family of graph convolutional layers is used to find the most active electrodes using the spatial position of electrodes. Finally, the corresponding EEG features of active electrodes are fed into the graph attention layers to detect auditory attention. The Fuglsang 2020 dataset is used in the experiments to train and test the proposed and baseline systems. The new TraGCNN approach, as compared with state-of-the-art attention classification methods from the literature, yields the highest performance in terms of accuracy (80.12%) as a classification metric. Additionally, the proposed model results in higher performance than our previously graph-based model for different lengths of EEG segments. The new TraGCNN approach is advantageous because attenuation detection is achieved from EEG signals of subjects without requiring speech stimuli, as is the case with conventional auditory attention detection methods. Furthermore, examining the proposed model for different lengths of EEG segments shows that the model is faster than our previous graph-based detection method in terms of computational complexity. The findings of this study have important implications for the understanding and assessment of auditory attention, which is crucial for many applications, such as brain–computer interface (BCI) systems, speech separation, and neuro-steered hearing aid development.

## 1. Introduction

The study of attention in science starts in psychology, where thorough behavioral experiments can result in exact displays of the tendencies and capabilities of attention in various situations [1]. Attention is a core property of human cognitive operations that mediates perception and behavior by focusing sensory and cognitive resources on related information in the stimulus space. Given our limited ability to process information, the attention mechanism selects, modulates, and focuses on the information most relevant to the situation [2].

In 1953, Colin Cherry started research on the cocktail party problem for the first time, describing how attention alters perception in a crowded setting with multiple sound sources [3]. The cocktail party phenomenon characterizes the brain’s ability to attend to a single speaker among many others, which could help separate a specific speech source more accurately from many sound sources. Although this is a highly complex cognitive problem, the human brain instantly solves this problem with compelling ease and accuracy that is difficult to match with any currently available algorithm [4].

Modeling auditory attention detection (AAD) in auditory scene analysis (ASA) has led to several promising applications. Research has shown the effectiveness of AAD in brain–computer interface (BCI) systems [2], controlling sound recording devices [5], robotics [6], and the sound separation of a single instrument in musical scenarios [7]. One particularly promising application is in neuro-steered hearing implants, where AAD can enhance the speech of a hearing-impaired (HI) individual in a situation with multiple speakers [8,9,10].

Various selective auditory attention detection (SAAD) methods have been suggested to determine the target speaker based on two approaches: (1) utilizing stimulus-response modeling, and (2) employing informative features from the brain and/or stimuli signals. The initial set of SAAD approaches starts with recognizing that the attended stream’s amplitude envelope influences neural responses [11,12,13,14,15,16,17]. The leading techniques for interpreting listeners’ attentional selection rely on estimating linear relationships between the characteristics of sound streams and EEG responses (forward mapping) or the opposite (backward mapping). Research has demonstrated that such mapping functions can be determined using the envelope of attended speech and EEG responses, which are then used to distinguish between attended and unattended speakers [13]. These discoveries have resulted in the development of one of the most commonly utilized SAAD techniques, known as temporal response functions (TRFs), which involves creating decoders to reconstruct the stimuli’s amplitude envelopes [18,19]. Due to the large data requirement, the TRF-based approaches are unsuitable for emerging real-time applications like intelligent hearing aid designs or brain–computer interfaces [20]. On the other hand, some researchers have conducted experiments to discern auditory attention by using key features in EEG signals and/or auditory stimuli and then training classifiers based on machine learning approaches [18]. In another study, an innovative Q-learning-based system was proposed for detecting attention dynamics and analyzing the temporal changes in the input signal [21]. The proposed dynamic SAAD model is addressed as a sequential decision-making challenge that is solved using recurrent neural network (RNN) and reinforcement learning techniques such as Q-learning and deep Q-learning. In [22], researchers used a joint convolutional neural network and long short-term memory (CNN-LSTM) solution to detect auditory attention. Here, the model takes EEG signals and spectrograms of the multiple speakers as input and classifies the attention to one of the speakers. Cai et al. [23] introduced an end-to-end CNN system based on the topological distribution of oscillatory cortical activity to detect the spatial attention of listeners. Here, the detection of auditory spatial attention is performed using spectro-spatial features extracted from the specific topographical map of alpha power signals. Qiu et al. [8] presented a system based on prototype training and a decoder, termed as EEGWaveNet, to improve auditory attention detection while reducing the classification attributes of the input signals. This model uses the energy of EEG in the time-frequency domain as input to better extract features related to auditory attention. A dense-net-based method for detecting auditory attention was proposed in [24]. Here, the authors transformed the two-dimensional (2D) EEG into a three-dimensional (3D) arrangement containing spatial–temporal information. Then, a special 3D deep convolutional neural network (DenseNet-3D) was employed to extract temporal and spatial features of the neural representation for the attended subjects.

In this paper, a novel selective auditory attention detection system is proposed using a transformer and a group of graph neural network layers. The proposed SAAD model, based on a transformer and a graph convolutional neural network, called TraGCNN-SAAD, employs a self-attention mechanism to emphasize important parts of the EEG signal in the attention detection procedure.

The key contributions of this paper are as follows:

1. Presenting of a hybrid end-to-end transformer and convolutional graph neural network for selective auditory attention detection;
2. Developing a transformer-based module to process sequences of EEG data responsible for temporal information of auditory attention; and
3. Incorporation of spatial module, including GraphSAGE, GCN, and graph attention layers to find important EEG electrodes for further processing, and consequently reduce the computational load of the attention detection.

The organization of the paper is as follows. Section 2 describes the methodology, including the data description and the proposed TraGCNN-SAAD model. Section 3 describes the experimental setup and evaluation procedure. The results of the evaluations and comparisons with some baseline methods from the literature for attention detection are discussed in Section 4. Here, the performance of the proposed model for different time segments of EEG data is presented and compared with our previous graph-based study. Concluding remarks and some future perspectives are given in Section 5.

## 2. Materials and Methods

### 2.1. Data Description

In this work, the publicly available dataset is used for the assessment of the experiments [25]. The collection of Fuglsang 2020 data involved 44 participants (aged 51–76), with 22 subjects having hearing impairments and the rest having normal hearing (NH). Only EEG data from subjects with normal hearing were used in the experiments. The EEG experiments took place in an electrically shielded double-walled sound booth. During the experiments, the participants were seated comfortably and directed to focus their gaze on a crosshair displayed on a computer screen. The speech stimuli comprised two audiobooks, read by a male and a female speaker. Any extended silent periods in the speech stimuli were shortened to 450 milliseconds long. The audio files were divided into approximately 50 s trials. The speech streams were positioned at ±90° using non-individualized head-related transfer functions (HRTFs). Loudness matching was employed to capture EEG responses to the two speech streams, unaffected by consistent differences in the sound levels between the target and masker speech. During the EEG data recording, the subjects selectively listened to one of the two simultaneous speech streams or a single speech stream in quiet conditions. EEG data was captured using a BioSemi Active-Two system equipped with 64 scalp electrodes positioned by the international 10–20 standard. The EEG data was digitized at a sampling rate of 512 Hz. In some cases, EEG was also measured within the ear canals, but this ear EEG data were excluded from the analysis of the current study. One participant (subject 24) faced disruptions during recording, so we excluded this participant from our data. For NH participants, the criteria for inclusion required audiometric thresholds to be within 20 dB of normal hearing level (HL) for frequencies up to 2 kHz and within 35 dB HL for frequencies above 2 kHz. One NH listener displayed a dip in the audiogram at 8 kHz that was 40 dB HL on the left ear and 30 dB HL on the right ear. To prevent subjects with thresholds above the clinical standard of 20 dB HL from influencing the results, those subjects were excluded from the analysis.

### 2.2. Theoretical Background

In this section, we will begin by introducing the general structure, history, and types of graph neural networks used in the proposed attention detection model.

Graphs can serve as a general form for displaying data in graph-based representations. Data from various systems across different domains, such as protein–protein interactions and brain networks, can be represented in graph structures. Furthermore, many real-world problems can be effectively modeled as processing on a small group of graphs, such as identification of different brain connections and prediction of the association between brain connection sub-networks and corresponding diseases [26,27].

A graph is described according to the following formula [27]:

(1) G=(V,E),

where V depicts the node or nodes of the graph, and E represents the edges. Nodes are the main components of a graph. In social networks, users are considered as nodes, whereas in chemical compounds, this role is taken by atoms. The number of nodes in a graph determines the order of a graph, and the set of edges represents the connections of nodes in the graph. Nodes $\nu_{1}$ and $\nu_{2}$ are assumed neighbors if an edge connects them. A graph can also be represented by an adjacency matrix that represents the connections between nodes. The adjacency matrix of the graph *G* is an N×N matrix, where *N* is equal to the number of nodes. We show the entry *i* and entry *j* of the matrix A as $A\left(i,j\right)$, showing the connection between two nodes $\nu_{i}$ and $\nu_{j}$. Specifically, if $A\left(i,\;j\right)=1$, it means there is an edge between two nodes, and if A(i,j)=0, there is no edge between them.

A subgroup of spectral clustering and dimensionality reduction techniques based on graph representations were among the early-stage machine learning approaches based on graph representations [26,27,28,29,30]. The techniques used in graph-based dimensionality reduction can be directly applied to learn the characteristics of nodes. *Word2vec* is one such method that could successfully separate words in a text [31]. Following the successes achieved in deep neural networks (DNNs), many attempts have been made to extend deep learning techniques to graphs. Graph neural networks (GNNs) have been developed due to these efforts, and are generally categorized into spectral and spatial methods [26]. In this regard, spectral-based techniques use filters established from the perspective of graph signal processing. Essentially, a basis is created through the eigen-decomposition of the graph Laplacian matrix. Then, node features are extracted for the model to use. In contrast, spatial-based approaches enhance traditional Euclidean convolution, such as the 2D convolution used in CNNs, by combining information from neighboring nodes. These convolutions are appealing due to their reduced computational complexity, localized characteristics, and transferability [29]. Since graphs are a universal way to represent data, GNNs are used in many fields, including natural language processing (NLP), computer vision, data mining, and medical care [30].

The standard convolution operations in convolutional neural networks (CNNs) [32] for images or text cannot be applied directly to graphs. The reason for this is the lack of coherent, specific, and fixed network structures on the graphs [33]. Similar to the convolution function of a typical CNN on an image, graph spatial methods define graph convolution based on the spatial relationships between nodes. In this context, images can be viewed as a special type of graph, where each pixel represents a node in the overall graph (i.e., the entire image). Graph convolutional neural networks (GCNNs) are closely related to graph recurrent neural networks (GRNNs). GRNNs generalize traditional RNNs to process the graph-structured data. They progressively evolve the multi-level node representations by stochastically merging two adjacent nodes with high compatibility [34]. Unlike GRNNs, which repeat node states, GCNNs architecturally represent cyclic interdependencies using a fixed number of layers with learnable weights in each layer [34]. Since the convolution of graphs with other neural networks is convenient and efficient, the popularity and development of these types of networks have increased dramatically in recent years. Since GCNNs have bridged the gap [29] between spectral and spatial-based approaches, spatial-based methods have undergone significant advances due to their greater efficiency, flexibility, and generalization. Neural networks for graphs (NN4G) [35] are considered one of the first efforts to use CNNs for spatial graphs. Unlike GRNNs, NN4Gs learn the interdependencies between nodes through a hybrid neural architecture with independent parameters in each layer. The neighborhood of a node can be expanded gradually through the construction of the graph architecture. NN4Gs perform graph convolution by directly summing the neighborhood information of each node. They also apply to skip connections to remember the information at each layer, similar to GCNN behavior. A critical difference between NN4G and GCNN is that it uses a non-normalized adjacency matrix that results potentially in different scaling of the node’s hidden states.

The transformer is a model architecture that avoids iteration and relies entirely on the attention mechanism to capture general dependencies between inputs and outputs [36]. Prior to the development of transformers, the dominant sequence-to-sequence models were based on complex recurrent or convolutional neural networks, which included an encoder and a decoder. Transformers also use an encoder and decoder, but they rely on attention mechanisms to allow for much greater parallelism than methods such as RNNs and CNNs. Models based on transformers, like bidirectional encoder representations from transformers (BERT) [37], or other variants of the transformer, provide high-accuracy results in many NLP tasks [38]. One of the primary differences between transformers and neural networks, such as RNNs, is multi-head attention. Multi-head attention is a module that applies an attention mechanism multiple times in parallel, allowing the model to weigh different aspects of input simultaneously. Then, the outputs of each independent attention head are concatenated and linearly transformed to the expected dimension. It uses self-attention and fully connected point layers for the encoder and decoder. Six identical layers are used in the encoder layer, each with two sub-layers. The first sublayer is a multi-head self-attention mechanism, and the second is a simple feed-forward network fully coupled to position direction. For the decoder layer, the same six layers as in the encoder are used, with the difference being that in addition to the two sub-layers found in each encoder layer, the decoder has a third sub-layer that performs multi-head attention on the output of the encoder group [36].

### 2.3. Related Works

Few studies have been conducted on auditory attention detection using graph-based methods. A graph-based approach was introduced in [16] for detecting selective auditory attention from the EEG signals using brain effective connectivity and complex network analysis. Here, listeners were divided into two groups of subjects based on whether they were focusing on the left or right ear, using artifact-free EEG signals of 60 s in duration. First, the connectivity matrices of all listeners were derived from the EEG data with the Granger causality method. Then, various features were extracted from these brain connectivity matrices. Finally, an optimized set of features was identified through feature selection and optimization techniques for training a classifier.

In [17], Cai et al. proposed a spiking graph convolutional network, called SGCN, that captures the spatial features of multi-channel EEG in a biologically plausible manner with low power consumption. This model translates brain signals into spikes and operates in an event-driven fashion with binary spike processing (1, 0), resulting in reduction of computation to just floating-point addition. The SGCN model is designed for low-power usage by only activating neurons when a certain number of input spikes exceed a specified threshold. Neurons that remain inactive can be switched to a low-power state.

### 2.4. Proposed Selective Auditory Attention Detection Model

**Figure 1:**
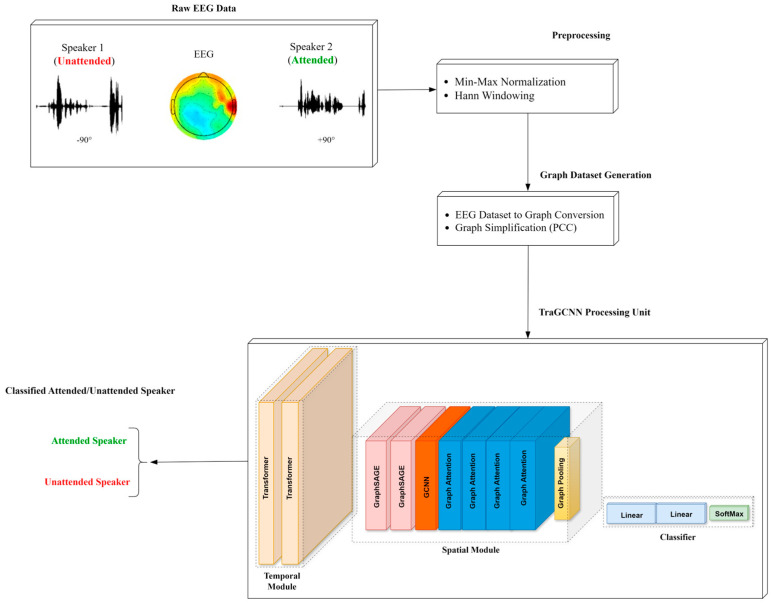
The block diagram of the proposed end-to-end TraGCNN-SAAD system for the detection of auditory attention. The system consists the modules for preprocessing and TraGCNN. Preprocessing aims to create a graph-based dataset. The new TraGCNN module consists of a transformer and a family of GCNNs for detecting auditory attention from the generated graph dataset.

In this study, we propose a new end-to-end system for selective auditory attention detection based on a transformer and GCNN. The proposed TraGCNN-SAAD system is shown in Figure 1.

First, the raw EEG data is fed into the preprocessing stage to normalize the input data and then window the signal with the Hann window. The windowing process is performed to evaluate the performance of the proposed system with different time segments of EEGs. In the next stage, called graph-based dataset generation, first, the normalized EEG data segments are converted to graphs. We use electrodes as nodes and connections between electrodes as edges for the graphs. Then, a graph simplification process is performed using the statistical method of Pearson correlation coefficients (PCC) to remove unimportant edges from the graph. This process is important to reduce the computational time required to train the model. The last stage of the proposed system concerns training the TraGCNN module based on the created graph dataset. This stage of the proposed system consists of a transformer and a family of GCNNs for processing the temporal and spatial dimensions of graph data, respectively.

#### 2.4.1. Graph Dataset Generation

Using the TorchEEG toolbox [39], first, a graph is constructed based on the EEG data channels and the standard 10–20 placement of 64 electrodes. TorchEEG is a library built on PyTorch for EEG signal analysis. Here, the electrodes are assumed as nodes, and the connections between them as edges for the graphs.

Correlation is a statistical measure that assesses the strength and direction of the linear association between two quantitative variables. Accordingly, in the next stage, a graph simplification technique is employed using the Pearson correlation coefficient (PCC) metric. The primary aim of this step is to reduce the computational load for the subsequent processing stages. Since not all nodes in the graph hold equal significance for the SAAD system, the less important nodes could carefully be eliminated using this metric. PCC can be calculated as follows [40]:

(2) $$r_{xy}=\frac{n\sum x_{i}y_{i}-\sum x_{i}\sum y_{i}}{\sqrt{n\sum x_{i}^{2}-(\sum x_{i}{)}^{2}}\sqrt{n\sum y_{i}^{2}-(\sum y_{i}{)}^{2}}},$$

where $r_{xy}$ denotes PCC, n is the sample size, and $x_{i}$ and $y_{i}$ are the individual sample points indexed with i. Here, $r_{xy}$ is calculated for each pair of nodes. If two nodes have $r_{xy}>0.7$, the two nodes and their corresponding edge are preserved; otherwise, they are removed from the graph, implying that they do not have information important enough for the attention mechanism in the TraGCNN processing unit.

#### 2.4.2. TraGCNN Processing Unit

**Figure 2:**
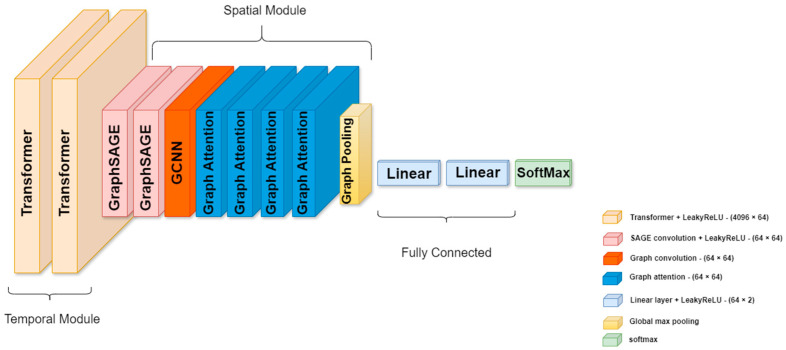
The proposed TraGCNN model. The model consists of two distinct parts. The first part consists of transformer layers which extract attentional information in the temporal domain, and the second part consists of a group of GraphSAGE, GCNN, and Graph Attention layers, which consider the position of important electrodes for detecting attention in the spatial domain.

The proposed TraGCNN extracts the temporal and spatial modalities of EEG data, respectively, as shown in Figure 2. The temporal module processes EEG data from remaining electrodes computed from PCCs. Here, auditory attentional information is extracted from time sequences of EEG data. In the spatial module, the significance of EEG electrodes in detecting attention is taken into account. Here, first, a full map of electrode positions is created based on PCC values of node connections and the corresponding time sequences of each node obtained in the temporal module. Then, a weighting coefficient is assigned to each connection of nodes based on its significance in relation to the whole graph.

Each module employs specific layer types tailored to process that particular modality. We hypothesize that dividing the TraGCNN unit into temporal and spatial modules based on data properties will result in higher detection accuracy, as each module will detect its specific modalities associated to the attention information from EEG data.

The first part of the proposed TraGCNN consists of the transformer layers and activation functions. The standard transformer model consists of two main components: an encoder and a decoder, each with a series of layers [41]. Each encoder layer contains an attention sublayer followed by a feedforward sublayer. The decoder has two attention sublayers, one for self-attention and one for cross-attention. An important part of the transformer network is a self-attention mechanism that allows a model to learn how to concentrate on relevant parts of input or output sequences [42]. This mechanism computes a weighted total of all inputs in a series, using weights based on the similarity between each item and a query vector. Self-attention is defined as [36]:

(3) $$Attention(Q,K,V)=SoftMax\left(\frac{QK^{T}}{\sqrt{d_{k}}}\right),$$

where **Q**, **K**, and **V** are query, key, and value matrices, respectively, and $d_{k}$ is the dimension of the query and key matrices. In the proposed system, **K** and **V** represent electrodes and their corresponding EEG data. Self-attention can learn long-distance connections, overall context, and sequences of varying lengths without relying on recurrence or convolution [43]. The formulation for the encoder and decoder is defined as [38,43]:

(4) $$z_{i}=\mathrm{EncoderLayer}(x_{i},Z_{<\;i}),$$

in which $x_{i}$ is an input and Z is a sequence of hidden states $z_{i}$ for input index i, and

(5) $$s_{j}=\mathrm{DecoderLayer}\left(y_{j},S_{<\;j},Z\right),$$

where $y_{j}$ is an output and S is a sequence of hidden states $s_{j}$. The decoder utilizes an attention mechanism to focus on the hidden states of the encoder. In the network designed for this study, two transformer layers are used to extract the time dimension characteristics of brain signals that contain attention cues in sequences. Their multi-head attention mechanism allows for dynamic selection and processing of critical data segments, enhancing the model’s ability to focus on relevant temporal features.

**Figure 3:**
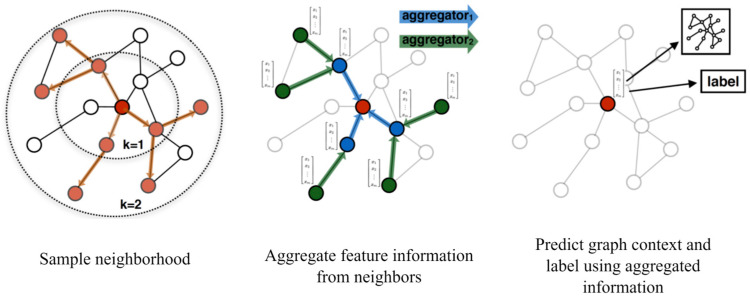
GraphSAGE operator. The key idea behind GraphSAGE is that the model learns how to aggregate feature information from a node’s local neighborhood [44,46].

The second part of the TraGCNN model (i.e., spatial module) consists of GNN-based layers. Here, first, the GraphSAGE (SAmple and aggreGatE) layers (see Figure 3) perform aggregation operations on graphs. The operations in the GraphSAGE layer are defined as follows [44,45]:

(6) $$x_{i}^{\prime }=W_{1}\;x_{i}+W_{2}\;.\;{\mathrm{mean}}_{j\;\in \;N(i)}\;x_{j},$$

where ‘mean’ represents the aggregator operator, $x_{i}$ is the input node feature, $x_{j}$(j ∈ N(i)) are features of neighborhood nodes, $W_{1}$ and $W_{2}$ are weight matrices, and $x_{i}^{\prime }$ is the result of applying the operator on $x_{i}$. In the designed model, two layers of GraphSAGE learn the spatial features of the EEG electrode placements using their aggregation operations.

After the data passes through GraphSAGE layers, the signal is fed into a GCNN [33] layer. While the GraphSAGE layers consider the local structure of individual graph nodes, the GCNN layer takes the global structure of the graph network into account. The GCNN layer processes the spatial information in the data and creates a map of overall electrode locations. This layer performs convolution operations on graph data. The convolution operation on graphs is defined as follows [29]:

(7) $$H^{\left(l+1\right)}=\sigma \left({\tilde{D}}^{-1/2}\;\tilde{A}\;{\tilde{D}}^{-1/2}H^{\left(l\right)}\;W^{\left(l\right)}\right),$$

where $\tilde{A}=A+I_{N}$ represents the adjacency matrix of the undirected graph G with *N* nodes and added self-connections, ${\tilde{D}}_{ii}=\sum_{j=0}{\tilde{A}}_{ij}$ denotes the diagonal elements of the degree matrix $\tilde{D}$, $I_{N}$ is the identity matrix, $W^{\left(l\right)}$ is a layer-specific trainable weight matrix, $H^{\left(l\right)}\in {\mathbb{R}}^{N\times D}$ is the matrix of activations in the l-th layer, and σ(.) denotes an activation function of the layer (e.g., LeakyReLU).

The last stage of the spatial module is the graph attention layers. As all information from nodes is not equally important, graph attention layers are developed to ensure that the information of critical nodes has a greater impact on other nodes through higher weight coefficients. Graph attention operators are described as [47,48]:

(8) $$x_{i}^{\prime }=\left(\sum_{j\;\in \;N(i)}\alpha_{ij}W\;x_{j}\right),$$

where $\alpha_{ij}$ is the attention coefficient, W is the weight matrix, $x_{j}$ is the source node information, $x_{i}^{\prime }$ is the destination node information, and j ∈ N(i), where N(i) is some neighborhood of the node i in the graph. The attention coefficient $\alpha_{ij}$ is calculated as follows:

(9) $$\alpha_{ij}=\frac{\exp\left(\mathrm{LeakyReLU}\left(a^{T}\left[Wx_{i}||Wx_{j}\right]\right)\right)}{\sum_{k\;\in \;N(i)}\exp\left(\mathrm{LeakyReLU}\left(a^{T}\left[Wx_{i}||Wx_{k}\right]\right)\right)},$$

where a is the parameterized weight vector, *T* is the transpose operator, and || represents the concatenation symbol. The coefficient $\alpha_{ij}$ causes the features of the source node j have a greater effect on the destination node i. As activation function, LeakyReLU [49] is used in the proposed system:

(10) $$\mathrm{LeakyReLU}(x)=\left\{\begin{array}{cc} x, & \;\mathrm{if}\;\;x\geq 0\\ \left(\mathrm{negative}\;\mathrm{slope}\right)\times x, & \;\mathrm{otherwise}. \end{array}\right.$$

A multi-head attention mechanism is employed in [47] to enhance the stability of the self-attention learning process. Specifically, *K*-independent attention mechanisms perform the transformation, and their features are then concatenated, leading to the subsequent output feature representation:

(11) $$x_{i}^{\prime }=\overset{K}{\underset{k=1}{\left|\right|}}\sigma \left(\sum_{j\in N_{i}}\alpha_{ij}^{k}\;W^{k}\;x_{j}\right),$$

where || means concatenation, $\alpha_{ij}^{k}$ are attention coefficients calculated by the k-th attention mechanism, and $W^{k}$ is the corresponding input linear transformation’s weight matrix. Note that by assuming K=1, Equation (11) reduces to Equation (8).

## 3. Results

### 3.1. Experimental Setup

The EEG data of 22 normal hearing subjects was taken from the experiment previously published by Fuglsang et al. [25] for the experiments conducted in this work. One subject experienced disruption during data recording (subject 24). Thus, only data from the remaining 21 subjects were retained during the experiments. There are two labels in the created EEG dataset for the direction of attention: one for attending to the left (Speaker 1), and one for attending to the right (Speaker 2).

The amount of data plays a vital role in training neural networks. An insufficient amount of data causes the model not to be trained enough and not have enough accuracy to identify unseen data. For this reason, the K-fold cross-validation method [50] was used for training. In this method, the entire data is divided into K groups, and then data groups are used for network training, while the remaining group is used for testing. A four-fold cross-validation was employed to assess model performance.

Data are randomly arranged within groups. This method achieves acceptable results in network training with a limited amount of data. It ensures that all data are used to train the network while preventing the model from overfitting data.

Two experiments were conducted to assess the performance of the “TraGCNN-SAAD” auditory attention detection method. In the first experiment, the detection accuracy of the proposed model was compared with two recent attention detection baseline systems from the literature [8,16]. There are some major differences between the processing stages of the two baseline systems. In the first baseline system [16], denoted here as “SAADconnectivity”, an approach for detecting selective auditory attention based on brain effective connectivity and graph-based analysis is presented. Here, the connectivity matrices of all subjects are computed with the Granger causality approach, which is used to find an optimal feature set for training the classifier. The second baseline system [8] implements a prototype training approach as a neuroscience-based method for decoding auditory attention. This training strategy utilizes a decoder model named EEGWaveNet, which applies the wavelet transform to EEG data to better capture the EEG energy distribution. In this baseline, two versions of the EEGWaveNet decoder model, denoted as “EEGWaveNet-K1” and “EEGWaveNet-K10”, are used, where K is the number of decision windows to obtain prototype samples.

The second experiment concerned evaluating the effectiveness of the proposed method for different durations of EEG segments. The duration of EEG segments is crucial for real-time applications, such as monitoring brain activity in cognitive tasks. Using short durations of EEG in such applications ensures reliability in dynamic environments where quick decisions are necessary.

### 3.2. Performance Measures

As evaluation metrics, we used accuracy, precision, recall, and F1-score [51] to assess the performance of different models. Accuracy represents the percentage of cases properly categorized within the dataset. The accuracy formula is as follows:

(12) $$\mathrm{Accuracy}=\frac{\mathrm{TP}+\mathrm{TN}}{\mathrm{TP}+\mathrm{TN}+\mathrm{FP}+\mathrm{FN}},$$

where TP (true positive) denotes the number of correctly classified positive samples; TN (true negative) represents the number of correctly classified negative samples; FP (false positive) is an error in binary classification in which a test result incorrectly indicates the presence of a condition (such as a disease when the disease is not present); and FN (false negative) is the opposite error, where the test result incorrectly indicates the absence of a condition when it is present.

Precision, often referred to as positive predictive value, represents the ratio of relevant instances to the total retrieved instances. Precision is calculated as follows:

(13) $$\mathrm{Precision}=\frac{\mathrm{TP}}{\mathrm{TP}+\;\mathrm{FP}}.$$

Recall, also known as sensitivity, indicates the proportion of relevant instances that are successfully retrieved. This measure is computed as:

(14) $$\mathrm{Recall}=\frac{\mathrm{TP}}{\mathrm{TP}+\;\mathrm{FN}}.$$

The F1-score is understood as a harmonic mean of precision and recall and achieves a highest value of one and lowest value of zero. The contributions of precision and recall to the F1-score are balanced. The equation for calculating the F1-score is:

(15) $$F1-\mathrm{score}=2\times \frac{\mathrm{Precision}\times \mathrm{Recall}}{\mathrm{Precision}+\mathrm{Recall}}.$$

### 3.3. Simulation Results

We used the PyTorch library [52] and PyG [45] module to develop our model and trained it on a system with an Intel Core i7-6700HQ CPU, 32 GB of RAM, and an Nvidia GTX 970 graphics card, with the model trained on the GPU. The EEG data of Fuglsang et al. [25] were used for training and testing of the baselines and proposed methods. The specifications of the proposed model are listed in Table 1.

**Figure 4:**
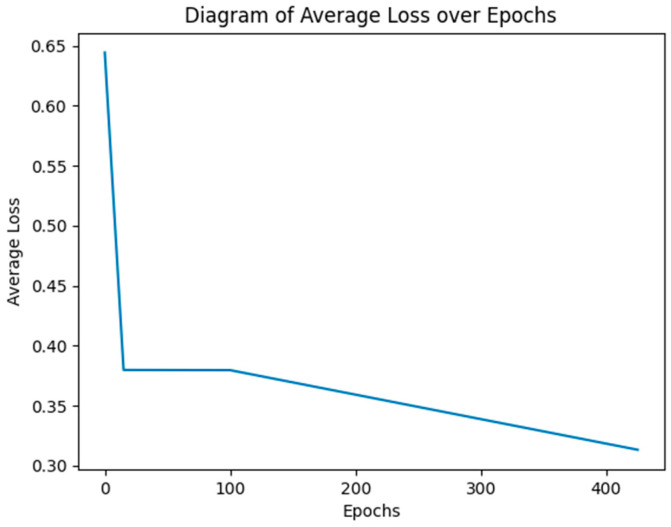
The plot of average loss with respect to epochs for the proposed model.

Table 2 shows the performance comparisons of different SAAD methods in terms of Accuracy, Precision, Recall, and F1-score. Among the baseline approaches, the “SAADconnectivity” method attained nearly the same attention detection results as those of the “EEGWaveNet-K1” and “EEGWaveNet-K10” models, despite the fact that this method uses an electrode reductions strategy in its implementation. Figure 4 indicates average loss reduction with training over epochs.

As in the case of “SAADconnectivity” method, the electrode reduction methodology was also employed in the proposed “TraGCNN-SAAD” approach using the PCC statistical metric. Nevertheless, the results of Table 2 verify that the proposed method still outperformed the previous state-of-the-art baselines. The reason for obtaining higher performance in the proposed model lies in using two different attention mechanisms, namely temporal and spatial modalities, for detecting auditory attention. The first mechanism employs self-attention in the *transformer* layers to extract attentional information from sequences of EEG data. The second attention detection mechanism uses *graph attention* layers to weigh the electrodes primarily responsible for attention detection. A reduction in electrodes could, in turn, reduce the computational load of the proposed method.

**Figure 5:**
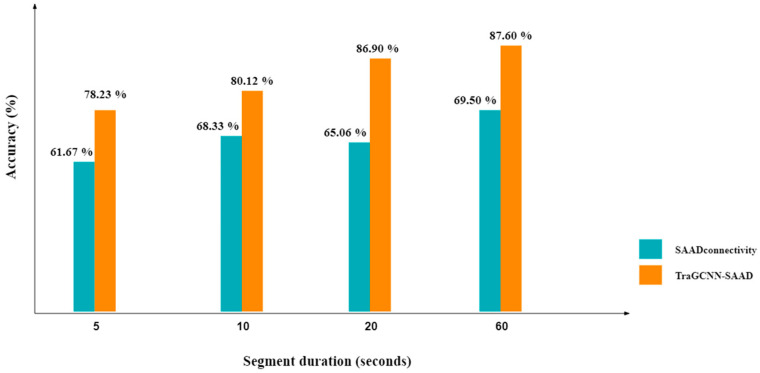
The performance of the proposed TraGCNN-SAAD and SAADconnectivity methods for different durations of EEG segments obtained by averaging the accuracy values for 4 folds of cross-validation. Each fold is composed of 15 epochs.

Finally, a further experiment was conducted to assess the effectiveness of the proposed “TraGCNN-SAAD” method for different durations of EEG segments. Figure 5 demonstrates the performance of the proposed SAAD method for various durations. Generally, the accuracy values decreased as the length of EEG data was shortened. Nonetheless, the findings indicate that the performance did not decrease significantly as the duration of EEG segments decreased, which is essential for real-time applications such as neuro-steered hearing devices.

## 4. Discussion

Two experiments were conducted to assess the performance of the “TraGCNN-SAAD” auditory attention detection system. In the first experiment, the proposed method’s performance was compared against different baseline SAAD approaches from the literature. The results verified that the proposed method outperforms the previous state-of-the-art baselines, despite using an electrode reduction strategy in its implementation. The reason for obtaining higher performance in the proposed system lies in using two different attention mechanisms, namely temporal and spatial modality, for detecting auditory attention. The second experiment evaluated the effectiveness of a proposed method using different durations of EEG data. EEG durations are important for real-time applications, such as tracking brain activity during cognitive tasks, as using shorter and reliable EEG segments ensures the model’s applicability in dynamic environments that require quick decision-making. It was expected that the model performance would deteriorate as the length of the EEG data shortened. However, the results of this experiment showed that accuracy values did not decrease significantly with shorter EEG segments.

## 5. Conclusions

Attention is a crucial element of human cognitive function, shaping both perception and actions by directing sensory and cognitive resources toward relevant information in our surroundings. Given our limited capacity for information processing, the attention mechanism effectively identifies, adjusts, and focuses on the most significant stimuli.

In this research, a new graph- and transformer-based end-to-end system is developed for detecting auditory attention, which shows significant promise in improving the accuracy and efficiency of auditory attention detection from EEG signals. This innovative approach leverages the strengths of both transformers and graph convolutional neural networks to create a robust model that can effectively capture complex patterns and relationships in EEG data while achieving improved results with lower computational requirements. The proposed model achieves higher performance by utilizing two attention mechanisms: temporal and spatial. The first mechanism uses self-attention in transformer layers to extract attention information from EEG sequences. The second employs graph attention layers to emphasize electrodes for attention detection, which helps reduce the computational load. Future efforts will focus on addressing the challenges and limitations of current methods to improve the proposed TraGCNN-SAAD model. The present research employs an experimental approach (i.e., dichotic listening) where each ear is exposed to distinct sounds at the same time. In a future study, the authors intend to explore the suggested method under more realistic auditory attention detection conditions, such as a cocktail party scenario, where the detection task can be performed in a real environment with multiple speakers, noise, and reverberation. Furthermore, among the current models of transformers, the computational time of the transformer layers used in this research is still high. This is likely due to the use of both encoders and decoders in the structure of the transformer in the proposed system. The use of a decoder-only transformer design could reduce the computational load of the auditory attention detection system. Furthermore, recent developments in machine learning offer graph transformers as a new class of neural network model for graph-structured data. It is known that the collaboration between transformers and graph learning leads to strong performance and flexibility in various graph-based tasks. Using graph transformers as a replacement for conventional transformer layers could enhance the accuracy of the proposed model.

## Figures and Tables

**Table 1:** The TraGCNN parameters.

Parameter	Value
Learning rate	0.001
Activation function	LeakyReLU [49]
Optimizer	Adam [53]
Loss	Cross entropy
Batch size	64
Nr. Of Epochs	15 for each fold
Dropout rate	0.2 [54]

**Table 2:** The performance comparisons of different SAAD approaches (in terms of accuracy) for EEG time segments of 10 s obtained by averaging results for 4 folds of cross-validation. Each fold is composed of 15 epochs.

Method	Accuracy	Precision	Recall	F1-Score
SAADconnectivity (our previous model)	68.33%	0.46	1.00	0.63
EEGWaveNet-K1 [8]	68.73%	-	-	-
EEGWaveNet-K10 [8]	69.97%	-	-	-
TraGCNN-SAAD (proposed)	80.12%	0.79	0.87	0.81

## Data Availability

The original data presented in the study are openly available in [25].
